# Hypercapnia Impairs Na,K-ATPase Function by Inducing Endoplasmic Reticulum Retention of the β-Subunit of the Enzyme in Alveolar Epithelial Cells

**DOI:** 10.3390/ijms21041467

**Published:** 2020-02-21

**Authors:** Vitalii Kryvenko, Miriam Wessendorf, Rory E. Morty, Susanne Herold, Werner Seeger, Olga Vagin, Laura A. Dada, Jacob I. Sznajder, István Vadász

**Affiliations:** 1 Department of Internal Medicine, Justus Liebig University, Universities of Giessen and Marburg Lung Center (UGMLC), Member of the German Center for Lung Research (DZL), 35392 Giessen, Germany; Vitalii.Kryvenko@innere.med.uni-giessen.de (V.K.); Miriam.Wessendorf@innere.med.uni-giessen.de (M.W.); Rory.Morty@mpi-bn.mpg.de (R.E.M.); Susanne.Herold@innere.med.uni-giessen.de (S.H.); Werner.Seeger@innere.med.uni-giessen.de (W.S.); 2 The Cardio-Pulmonary Institute (CPI), 35392 Giessen, Germany; 3 Department of Lung Development and Remodeling, Max Planck Institute for Heart and Lung Research, 61231 Bad Nauheim, Germany; 4 Department of Physiology, David Geffen School of Medicine, University of California at Los Angeles, Los Angeles, CA 90095, USA; olgav@ucla.edu; 5 Veterans Administration Greater Los Angeles Healthcare System, Los Angeles, CA 90073, USA; 6 Division of Pulmonary and Critical Care Medicine, Feinberg School of Medicine, Northwestern University, Chicago, IL 60611, USA; lauradada@northwestern.edu (L.A.D.); j-sznajder@northwestern.edu (J.I.S.)

**Keywords:** carbon dioxide, hypercapnia, Na,K-ATPase, endoplasmic reticulum, sodium transport, protein oxidation, alveolar epithelium

## Abstract

Alveolar edema, impaired alveolar fluid clearance, and elevated CO_2_ levels (hypercapnia) are hallmarks of the acute respiratory distress syndrome (ARDS). This study investigated how hypercapnia affects maturation of the Na,K-ATPase (NKA), a key membrane transporter, and a cell adhesion molecule involved in the resolution of alveolar edema in the endoplasmic reticulum (ER). Exposure of human alveolar epithelial cells to elevated CO_2_ concentrations caused a significant retention of NKA-β in the ER and, thus, decreased levels of the transporter in the Golgi apparatus. These effects were associated with a marked reduction of the plasma membrane (PM) abundance of the NKA-α/β complex as well as a decreased total and ouabain-sensitive ATPase activity. Furthermore, our study revealed that the ER-retained NKA-β subunits were only partially assembled with NKA α-subunits, which suggests that hypercapnia modifies the ER folding environment. Moreover, we observed that elevated CO_2_ levels decreased intracellular ATP production and increased ER protein and, particularly, NKA-β oxidation. Treatment with α-ketoglutaric acid (α-KG), which is a metabolite that has been shown to increase ATP levels and rescue mitochondrial function in hypercapnia-exposed cells, attenuated the deleterious effects of elevated CO_2_ concentrations and restored NKA PM abundance and function. Taken together, our findings provide new insights into the regulation of NKA in alveolar epithelial cells by elevated CO_2_ levels, which may lead to the development of new therapeutic approaches for patients with ARDS and hypercapnia.

## 1. Introduction

Na,K-ATPase (NKA) is a heterodimeric enzyme and a member of the P-type ATPase family. NKA is located at the basolateral plasma membrane (PM) of polarized cells, where the primary function of the enzyme is to extrude three sodium ions while taking up two potassium ions per pump cycle in an ATP-dependent manner [1,2]. A functional NKA requires a catalytic α-subunit and a regulatory β-subunit [2]. Additionally, a γ-subunit has also been identified, which represents a family of single-span transmembrane proteins containing the FXYD motif that is not an integral part of the transporter but rather regulates the activity and membrane abundance of the enzyme [3,4]. In the alveolar epithelium of the lung, the activity of NKA creates an Na^+^ gradient that drives reabsorption of fluid from the alveolar space, which keeps the alveoli relatively “dry,” which is essential for an effective gas exchange. The catalytic α-subunit of the transporter contains the binding sites for Na^+^, K^+^, and ATP [2]. The NKA β-subunit, which is a type II membrane glycoprotein, has a pivotal role in delivery and appropriate insertion of the NKA-α subunit in the PM [1]. Remarkably, mice deficient in the NKA-β subunit in alveolar epithelial cells have reduced alveolar fluid clearance, which results in aggravation of acute lung injury (ALI) and further underlies the pivotal role of NKA-β in the overall transporter function [5]. Additionally, numerous reports have shown that the function of NKA-β is not limited to regulation of NKA-α, but is centrally involved in establishing epithelial cell polarity, formation of adherens junctions, and regulation of paracellular permeability, which are key for maintaining a functional epithelial barrier [6,7,8,9,10]. 

Carbon dioxide (CO_2_) is a byproduct of mitochondrial respiration and cellular metabolism. Excess of CO_2_, in mammals, is eliminated by the lungs under physiological conditions [11,12]. Thus, any condition that leads to alveolar hypoventilation or impairs diffusion of CO_2_ across the alveolar-capillary barrier results in retention of CO_2_ in the blood, which is termed hypercapnia. During ALI and in patients with acute respiratory distress syndrome (ARDS), disruption of the alveolar-capillary barrier, and, thus, accumulation of edema fluid in the interstitial and alveolar spaces, may result in hypercapnia. Moreover, hypercapnia is often further potentiated or even directly caused by protective ventilation strategies with low tidal volumes to limit further lung damage [12,13]. Remarkably, hypercapnia was found to decrease alveolar fluid clearance and resolution of alveolar edema by decreasing the PM abundance of the Na,K-ATPase [14,15,16]. Since ALI is often associated with hypercapnia and is characterized by alveolar edema and disruption of epithelial junctions [17], further understanding of the mechanisms impairing the NKA function and of the potential rescue mechanisms might be of critical importance promoting the resolution of epithelial injury, alveolar repair processes, and edema resolution.

About one-third of all cellular proteins interact with the endoplasmic reticulum (ER) during folding and maturation processes [18]. Of note, in the ER, the NKA-β undergoes various post-translational modifications, including glycosylation and assembly with NKA-α, before leaving the ER and, subsequently, being transferred to the PM [19,20]. Whether hypercapnia affects ER protein folding of NKA has not been previously investigated. In the current study, we explored how elevated CO_2_ levels influence the ER environment and expression, PM abundance, and function of the NKA-β subunit. Understanding the molecular mechanisms underlying the effects of hypercapnia on the folding and maturation of the NKA-β in the alveolar epithelium might provide new therapeutic strategies for the treatment of patients with ARDS and hypercapnia.

## 2. Results

### 2.1. Hypercapnia Increases the Endoplasmic Reticulum Fraction of the Na,K-ATPase β-Subunit

**Figure 1:**
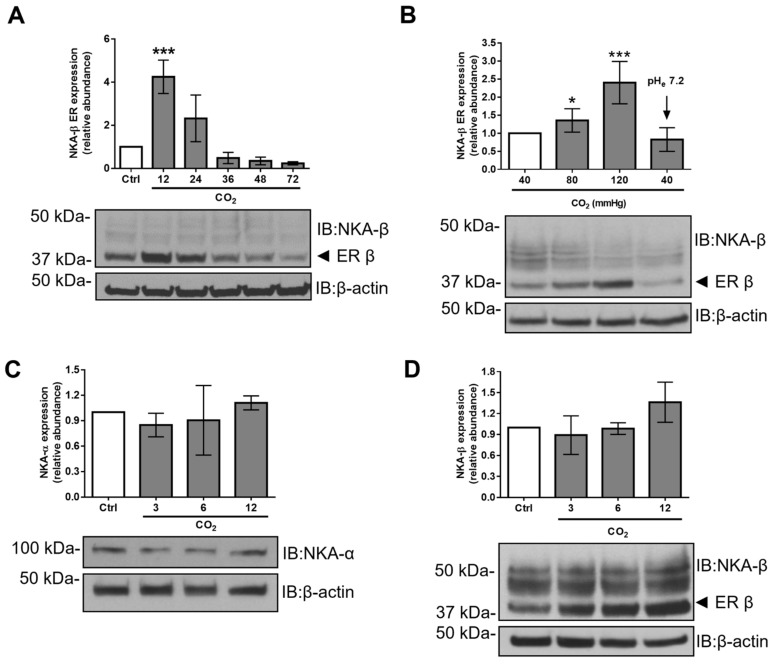
Hypercapnia increases Na,K-ATPase (NKA)-β abundance in the endoplasmic reticulum (ER). (**A**) A549 cells were exposed to 40 (Ctrl) or 120 mmHg CO_2_ (CO_2_) with an extracellular pH = 7.4 for the different time-points up to 72 h. Total cellular level of NKA-β was measured by immunoblotting. Representative immunoblots of NKA-β are shown. Bars represent ER-resident NKA-β/β-actin ratio. Values are expressed as mean ± SD (*n* = 3, *** *p* < 0.001). (**B**) A549 cells were treated with 40, 60, 80, and 120 mmHg of CO_2_ with an extracellular pH = 7.4 or to 40 mmHg CO_2_ with a pH = 7.2 for 12 h. NKA-β levels were measured by immunoblotting. Representative immunoblots of NKA-β are shown. Bars represent total NKA-β/β-actin ratio. Values are expressed as mean ± SD (*n* = 3, * *p* < 0.05, *** *p* < 0.001). (**C**) A549 cells were exposed to 40 (Ctrl) or 120 mmHg CO_2_ (CO_2_) with an extracellular pH = 7.4 for different time-points. Total cellular levels of NKA-α were measured by immunoblotting. Representative immunoblots of NKA-α are shown. Bars represent total NKA-α/β-actin ratio. Values are expressed as mean ± SD (*n* = 3). (**D**) A549 cells were exposed to 40 (Ctrl) or 120 mmHg CO_2_ (CO_2_) for different time-points. Total cellular levels of NKA-β were measured by immunoblotting. Representative immunoblots of NKA-β are shown. Bars represent total NKA-β/β-actin ratio. Values are expressed as mean ± SD (*n* = 3).

The NKA-β subunit is a glycoprotein that undergoes posttranslational maturation processing in the ER and Golgi prior to delivery to the plasma membrane. After the initial step of ER folding, the addition of an oligosaccharide core results in the formation of a specific high mannose N-glycan type NKA-β, which resides exclusively in the ER [19]. To investigate whether hypercapnia affects cellular levels of NKA-β, we exposed alveolar epithelial cells (AEC) for up to 72 h to physiological or increased levels of CO_2_ and analyzed the protein expression pattern of NKA-β and NKA-α (Figure 1A).

We observed a transient and time-dependent increase in the abundance of ER-resident NKA-β, which reached a maximum at 12 h and lasted for at least 24 h upon hypercapnic exposure. Our subsequent experiments were performed after a 12-h hypercapnia exposure, where the highest ER-resident NKA-β abundance was observed. To demonstrate whether this effect was directly driven by CO_2_ or the elevated CO_2_-associated acidosis, AEC were treated with increasing CO_2_ concentrations (up to 120 mmHg) with normal (pH = 7.4) or acidic (pH = 7.2) extracellular pH (Figure 1B). Of note, we observed that the acidic environment per se did not affect the levels of the ER-resident NKA-β. In addition, we did not find significant differences in the total protein levels of NKA-α and NKA-β subunits upon hypercapnic exposure for up to 12 h (Figure 1C,D).

### 2.2. Elevated CO_2_ Levels Decrease Na,K-ATPase Plasma Membrane Abundance and Function

**Figure 2:**
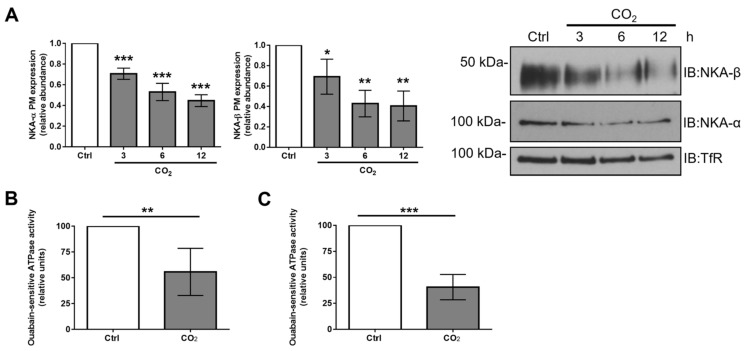
Exposure to elevated CO_2_ levels for up to 12 h decreases Na,K-ATPase plasma membrane abundance and function. (**A**) A549 cells were exposed to 40 (Ctrl) or 120 mmHg CO_2_ (CO_2_) for different time-points. Plasma membrane (PM) abundance of NKA-α and NKA-β was determined by biotin-streptavidin pull-down and immunoblotting. Representative immunoblots of NKA-α, NKA-β, and transferrin receptor (TfR) at the PM are shown. Bars represent the NKA-α or NKA-β/TfR ratio. Values are expressed as mean ± SD (*n* = 3, * *p* < 0.05, ** *p* < 0.01, *** *p* < 0.001). (**B**) Primary rat ATII and (**C**) human A549 cells were exposed to 40 (Ctrl) or 120 mmHg CO_2_ (CO_2_) with extracellular pH = 7.4 for 12 h. The PM fraction was isolated by ultracentrifugation and ouabain-sensitive NKA activity was measured by using a colorimetric ATP bioluminescence assay kit. Values are expressed as mean ± SD (*n* = 5, ** *p* < 0.01, *** *p* < 0.001).

It has been previously demonstrated that NKA-α cannot leave the ER before being assembled with the regulatory NKA-β subunit and that only a functional NKA α:β complex exported from the ER can reach the cellular surface [19,20]. Thus, to determine whether the hypercapnia-induced increase in the amount of ER-resident NKA-β influenced PM expression of the NKA, we performed a cell-surface protein biotinylation and the streptavidin pull-down assay (Figure 2A).

Exposure of AEC to elevated CO_2_ concentrations decreased the cell surface abundance of the NKA-α and NKA-β subunits in a time-dependent manner. These findings correlated with the above-mentioned increase in the levels of ER-resident NKA-β (Figure 1D), which suggests that the ER retention of NKA-β contributed to the decreased cell-surface expression of the enzyme. Next, NKA function was assessed by measuring ouabain-sensitive ATPase activity in isolated PM fractions from primary rat alveolar epithelial type II (ATII) and human A549 cells (Figure 2B,C). Consistent with the results of our biotinylation assays, in both cellular cultures, elevated CO_2_ levels markedly decreased ouabain-sensitive ATPase activity, which indicates reduced NKA function.

### 2.3. Hypercapnia Induces Endoplasmic Reticulum Retention of the Na,K-ATPase β-Subunit

**Figure 3:**
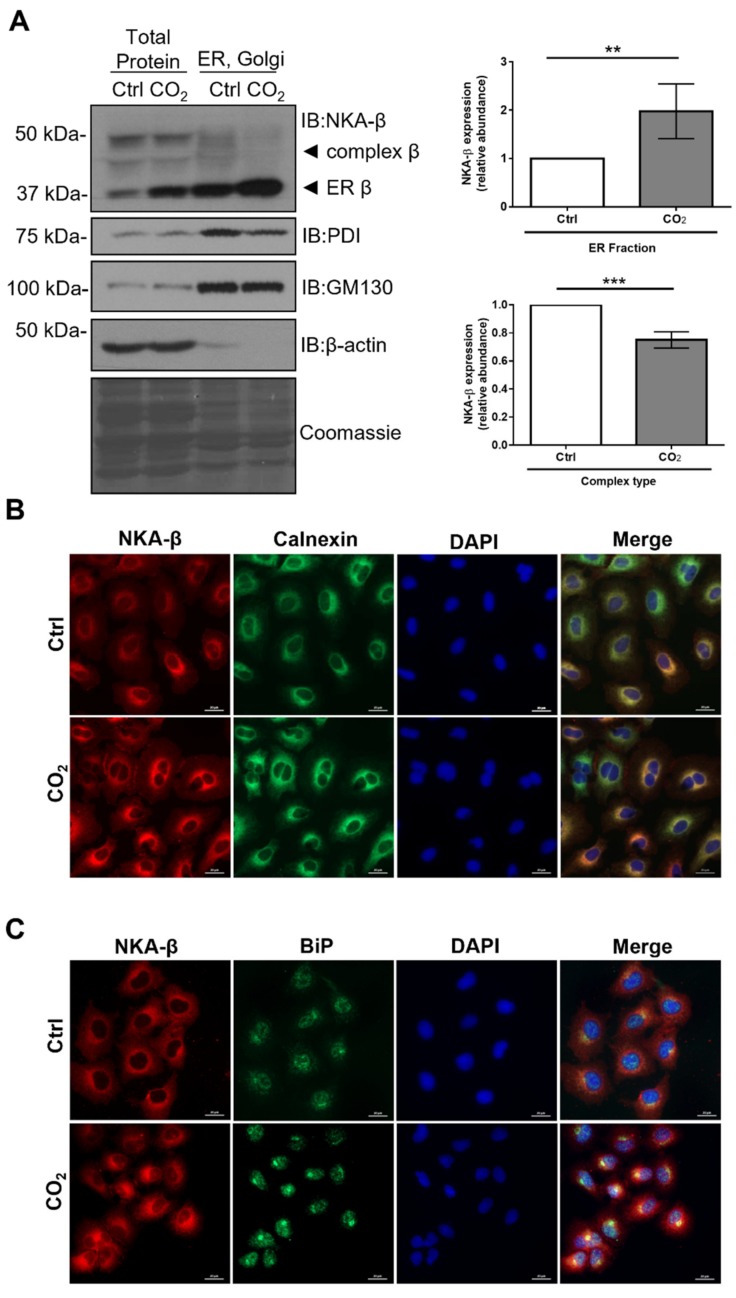
Elevated CO_2_ levels promote endoplasmic reticulum (ER) retention of Na,K-ATPase-β. (**A**) A549 cells were exposed to 40 (Ctrl) or 120 mmHg CO_2_ (CO_2_) with an extracellular pH = 7.4 for 12 h. Subcellular fractions were isolated by ultracentrifugation and protein levels of the NKA-β, protein disulfide isomerase (PDI; an ER marker), and Golgin subfamily A member 2 (GM130;a Golgi marker) were analyzed by immunoblotting. Representative Western blots are shown. Bars represent ER NKA-β/Coomassie ratio or complex type NKA-β/Coomassie ratio. Values are expressed as mean ± SD (*n* = 4, ** *p* < 0.01, *** *p* < 0.001). (**B**) A549 cells were exposed to 40 (Ctrl) or 120 mmHg CO_2_ (CO_2_) with an extracellular pH = 7.4 for 12 h. Cellular localization of NKA-β and calnexin were determined by immunofluorescence. Immunofluorescence staining of NKA-β (red), calnexin (green), and nuclei (blue) are shown. Scale bar—20 µM. (**C**) A549 cells were exposed to 40 (Ctrl) or 120 mmHg CO_2_ (CO_2_) with an extracellular pH = 7.4 for 12 h. Cellular localization of NKA-β and BiP were determined by immunofluorescence. Representative immunofluorescence staining of NKA-β (red), BiP (green), and nuclei (blue) are shown. Scale bar—20 µM.

The increased abundance of ER-resident NKA-β (that was not efficiently delivered to the plasma membrane) might be explained by retention of NKA-β in the ER [20]. To test this hypothesis, we performed isolation of ER and Golgi subcellular fractions from total cellular lysates and determined the amount of high mannose and complex N-glycan type NKA-β (Figure 3A). We observed increased amounts of NKA-β in the ER upon hypercapnia, which was associated with decreased Golgi-resident complex forms of the enzyme. These results suggested that newly synthesized forms of NKA were not processed to further maturation steps but were retained in the ER, which leads to decreased cell-surface abundance of the transporter.

Recent reports showed that the ER chaperones, binding immunoglobulin protein (BiP), and calnexin are required for the folding and retention of the NKA-β subunit upon normal and stress conditions [20]. To further assess if those chaperones were involved in the hypercapnia-induced ER retention of the NKA-β, AEC were exposed to normal or elevated CO_2_ levels and localization of NKA-β, calnexin, and BiP were detected by immunofluorescent microscopy (Figure 3B,C). Enhanced co-localization of NKA-β with calnexin and BiP was observed upon hypercapnia, which indicates that both chaperons might be involved in the process of ER retention.

### 2.4. Hypercapnia Attenuates Na,K-ATPase α:β Complex Formation

**Figure 4:**
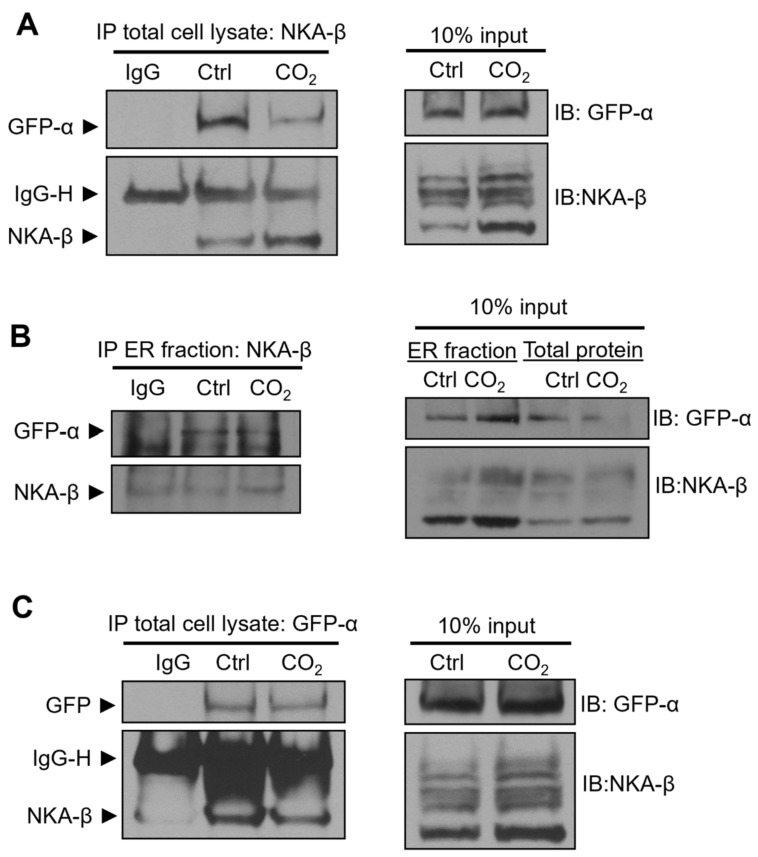
Elevated CO_2_ levels decrease formation of the Na,K-ATPase-α:β complex. (**A**) A549-α_1_-GFP expressing cells were exposed to 40 (Ctrl) or 120 mmHg CO_2_ (CO_2_) with an extracellular pH = 7.4 for 12 h. NKA-β was immunoprecipitated from the whole cell lysate by using an NKA-β-specific antibody and levels of co-immunoprecipitated NKA-α were analyzed by immunoblotting. Representative Western blots are shown (*n* = 4). (**B**) A549-α_1_-GFP expressing cells were exposed to 40 (Ctrl) or 120 mmHg CO_2_ (CO_2_) with an extracellular pH = 7.4 for 12 h. The ER fraction was isolated and NKA-β was immunoprecipitated and the levels of co-immunoprecipitated NKA-α were analyzed by immunoblotting. Representative Western blots are shown (*n* = 3). (**C**) A549-α_1_-GFP expressing cells were exposed to 40 (Ctrl) or 120 mmHg CO_2_ (CO_2_) with an extracellular pH = 7.4 for 12 h. NKA-α_1_-GFP (GFP-α) was immunoprecipitated from the whole cell lysate by using a GFP-specific antibody and the levels of co-immunoprecipitated NKA-β were analyzed by immunoblotting. Representative Western blots are shown (*n* = 4).

Previous studies have reported that ER quality control allows only export of assembled NKA α:β complexes at a 1:1 stoichiometric ratio to the Golgi, which sustains an equimolar ratio of NKA α-subunits and β-subunits at the cellular surface [19,21]. Having demonstrated that elevated CO_2_ levels caused ER retention of the NKA-β, we further analyzed the formation of the NKA α:β complex upon hypercapnia treatment. To this end, we employed A549-α_1_-green fluorescent protein (A549-α_1_-GFP) cells in which GFP was fused to the NKA-α subunit and exposed those cells to normal or elevated CO_2_ levels. Afterward, we immunoprecipitated NKA-β from either total cell lysates or ER fractions by using specific antibodies and the amount of co-immunoprecipitated NKA-α was detected by immunoblotting (Figure 4A,B). Our results showed that hypercapnia decreased the amount of precipitated NKA α-subunit, which suggests that the ER-retained NKA-β was only partially assembled with NKA-α. Similar results were obtained when reverse co-immunoprecipitation with an antibody against GFP was performed (Figure 4C).

### 2.5. Elevated CO_2_ Levels Alter the Oxidizing Environment of the Endoplasmic Reticulum and Promote Oxidation of the Na,K-ATPase β-Subunit

**Figure 5:**
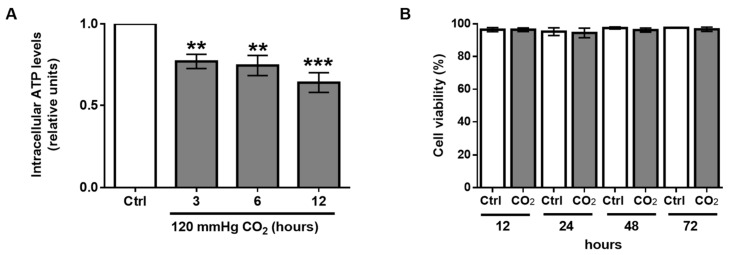
Hypercapnia decreases intracellular ATP production but does not affect cell viability. (**A**) A549 were treated with normal (Ctrl, 40 mmHg, pHe = 7.4) or elevated CO_2_ levels (CO_2_, 120 mmHg, pHe = 7.4) for different time-points. Intracellular ATP levels were measured by using an ATP bioluminescence assay kit. Bars represent total ATP/total protein ratio. Values are expressed as mean ± SD (*n* = 3, ** *p* < 0.01, *** *p* < 0.001). (**B**) A549 cells were exposed to normal (Ctrl, 40 mmHg, pHe = 7.4) or elevated CO_2_ levels (CO_2_, 120 mmHg, pHe = 7.4) for different time-points. Cell viability was measured by assessing the plasma membrane integrity. Graph bars represent the percentage of viable cells. Values are expressed as mean ± SD (*n* = 3).

It is well documented that a fully functional ER is dependent on physiological levels of Ca^2+^ and ATP and requires an oxidizing environment [22,23]. However, it has been shown that sustained hypercapnia and hypercapnic acidosis may decrease ATP production and perturb mitochondrial function [24,25]. Thus, we speculated that elevated CO_2_ levels may prevent NKA-β maturation in the ER by altering the metabolic status of the cell. In line with this notion, we observed a significant decrease in intracellular ATP levels in hypercapnia-exposed AEC (Figure 5A). Of note, cell viability was not affected by elevated CO_2_ levels even when AEC were exposed to hypercapnia for up to 72 h (Figure 5B).

**Figure 6:**
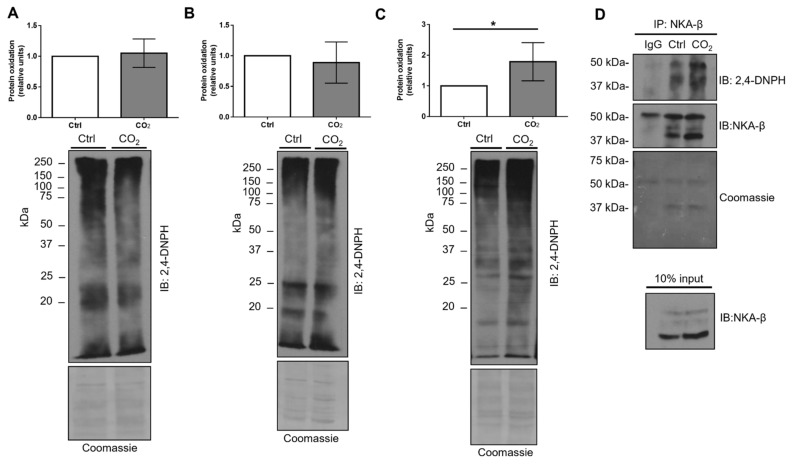
Hypercapnia increases ER protein and Na,K-ATPase-β oxidation levels. A549 cells were exposed to 40 (Ctrl) or 120 mmHg CO_2_ (CO_2_) with an extracellular pH = 7.4 for 12 h. Next, cellular fractions were isolated by ultracentrifugation and protein oxidation, which was determined in (**A**) total protein, (**B**) cytosolic, and (**C**) ER fraction by immunoblotting against 2,4-dinitrophenylhydrazone (2,4-DNPH). Bars represent a 2,4-DNPH/Coomassie ratio. Values are expressed as mean ± SD (*n* = 5, * *p* < 0.05). (**D**) A549 cells were exposed to normal (Ctrl, 40 mmHg, pHe = 7.4) or elevated CO_2_ levels (CO_2_, 120 mmHg, pHe = 7.4) for 12 h. NKA-β was then immunoprecipitated and protein oxidation was determined as described above. Representative Western blots are shown.

It has been previously reported that decreased cellular ATP levels can promote protein oxidation by activating redox reactions [26,27]. Therefore, we next hypothesized that hypercapnia may alter the oxidizing environment of cells. To determine whether increased CO_2_ levels affect protein oxidation in hypercapnia-treated AEC, we isolated total, cytosolic, and ER fractions from whole-cell homogenates by ultracentrifugation and, subsequently, assessed protein oxidation in these fractions. Importantly, in contrast to the total protein and cytosolic fractions (Figure 6A,B), hypercapnia treatment augmented the levels of protein oxidation in the ER (Figure 6C).

In addition, to determine whether NKA was a substrate of protein oxidation, AEC were exposed to normal or elevated CO_2_ concentrations and the levels of protein oxidation in immunoprecipitated NKA-β were detected by immunoblotting (Figure 6C). Of note, we detected that the levels of NKA-β oxidation were increased when exposed to elevated CO_2_ levels, which suggests that hypercapnia dysregulated the oxidizing status of the ER environment. Therefore, it induces the oxidation of NKA-β and potentially disrupts normal maturation of the enzyme.

### 2.6. Treatment with α-Ketoglutaric Acid Ameliorates Hypercapnia-Induced ER Dysfunction and Restores Na,K-ATPase Function

**Figure 7:**
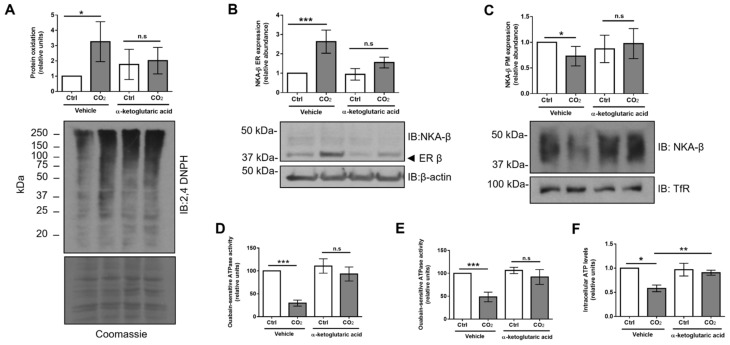
Treatment with α-KG reverses hypercapnia-induced protein oxidation and decreases ER-retained forms of Na,K-ATPase. (**A**) A549 cells were exposed to 40 (Ctrl) or 120 mmHg CO_2_ (CO_2_) with an extracellular pH = 7.4 for 12 h in the presence or absence of α-KG (10 mM) or vehicle. The ER fraction was isolated by ultracentrifugation and the level of protein oxidation was assessed as described above. Bars represent 2,4-DNPH/Coomassie ratio. Values are expressed as mean ± SD (*n* = 3, * *p* < 0.05, n.s—non-significant). (**B**) A549 cells were exposed to 40 (Ctrl) or 120 mmHg CO_2_ (CO_2_) with an extracellular pH = 7.4 for 12 h in the presence or absence of α-KG or vehicle. NKA-β levels were measured by immunoblotting. Representative immunoblots are shown. Bars represent the ER-resident NKA-β/β-actin ratio. Values are expressed as mean ± SD (*n* = 5, *** *p* < 0.001, n.s. – non-significant). (**C**) A549 cells were exposed to 40 (Ctrl) or 120 mmHg CO_2_ (CO_2_) for 12 h in the presence or absence of α-KG or vehicle. NKA-β PM abundance was determined by biotin-streptavidin pull-down and immunoblotting. Representative immunoblots of NKA-α, NKA-β, and TfR at the PM are shown. Bars represent the NKA-β/TfR ratio. Values are expressed as mean ± SD (*n* = 5, * *p* < 0.05, n.s—non-significant). (**D**) Primary rat ATII and (**E**) A549 cells were exposed to 40 (Ctrl) or 120 mmHg CO_2_ (CO_2_) with extracellular pH = 7.4 for 12 h in the presence or absence of α-KG or vehicle. The PM fraction was isolated by ultracentrifugation and ouabain-sensitive Na,K-ATPase activity was measured by using a colorimetric ATP bioluminescence assay kit. Values are expressed as mean ± SD (*n* = 5, *** *p* < 0.001, n.s. – non-significant). (**F**) A549 cells were exposed to 40 (Ctrl) or 120 mmHg CO_2_ (CO_2_) with an extracellular pH = 7.4 for 12 h in the presence or absence of α-KG or vehicle. Intracellular ATP levels were measured by using an ATP bioluminescence assay kit. Bars represent total ATP/total protein ratio. Values are expressed as mean ± SD (*n* = 3, * *p* < 0.05, ** *p* < 0.01).

Up to this point, our studies have suggested that hypercapnia enhances retention of ER-resident NKA-β due to increased protein oxidation in the ER. In a recent publication, it has been shown that elevated CO_2_ levels inhibit expression of isocitrate dehydrogenase 2 (IDH2), which is a key enzyme in the tricarboxylic acid (TCA) cycle. Thereby, it causes mitochondrial dysfunction and ATP depletion [24]. Furthermore, treatment with α-ketoglutaric acid (α-KG) compensated the loss of IDH2 and raised ATP levels in hypercapnia-exposed cells [24]. Thus, we hypothesized that the increased ER oxidation by elevated CO_2_ levels was due to mitochondrial dysfunction that might be rescued by administration of α-KG. Treatment of AEC with α-KG prevented the hypercapnia-induced increase in protein oxidation in the ER (Figure 7A).

Next, we determined the effects of α-KG treatment on ER-resident and PM-located NKA-β upon hypercapnia. AEC were exposed to normal or elevated levels of CO_2_ for 12 h in the presence or absence of α-KG and, subsequently, the ER faction and cell surface abundance of NKA-β were measured (Figure 7B,C). Our results revealed that α-KG treatment rescues the effects of hypercapnia on the levels of NKA-β in the ER, which was associated with an increased expression of NKA-β at the PM. Moreover, to determine whether an increased cell surface abundance of the NKA-β resulted in an elevation of NKA activity, we measured ouabain-sensitive ATPase activity in primary and cultured AEC (Figure 7D,E) and observed that treatment with α-KG partially restored NKA function upon hypercapnia. Lastly, treatment with α-KG partially restored the hypercapnia-induced reduction in total intracellular ATP levels (Figure 7F). Taken together, these studies suggest that rescuing mitochondrial function and administration of α-KG restore the hypercapnia-induced ER retention of NKA-β, which increases PM abundance and activity of NKA.

## 3. Discussion

In the present study, we show that, in alveolar epithelial cells, hypercapnia downregulates NKA function by promoting oxidation of NKA-β, which, thereby, impairs its interaction with NKA-α and retains the misfolded NKA-β in the ER. This decreases PM abundance and activity of the transporter. In addition, we demonstrate that treatment with α-KG, which is an active metabolite, that has been previously shown to rescue the impaired TCA cycle and ATP production in hypercapnia-exposed cells, attenuates the elevated CO_2_–induced NKA-β oxidation in the ER, which increases the abundance of the enzyme at the PM.

Effective alveolar gas exchange requires relatively “dry” airspaces achieved by an active vectorial Na^+^ transport process across the alveolar epithelium [28], driven by the basolaterally-located NKA and the apical epithelial sodium channel, ENaC, which promotes alveolar fluid clearance [17,29,30]. It is well documented that impaired function/expression of these transporters have a deleterious impact on the resolution of lung edema in models of ALI [31,32]. In patients with ARDS, disruption of the alveolar-capillary barrier causes pulmonary edema that severely alters gas exchange and leads to hypoxia and hypercapnia. Since patients with ARDS require ventilation with low tidal volumes to limit ventilator-induced lung injury, hypercapnia is often further aggravated in this patient group [11,12], which has been tolerated in the last two decades. This is a concept termed “permissive hypercapnia.” Hypercapnia is a double-edged sword and the associated acidosis may exhibit advantageous anti-inflammatory effects [33]. However, recent reports suggest that, in patients with severe hypercapnia, elevated levels of CO_2_ are associated with higher complication rates, more organ failure, worse outcome, and increased risk of intensive care unit mortality [34]. A possible explanation, in addition to the recently established negative effects of elevated CO_2_ levels on innate immunity and host defense [35,36,37], might be the hypercapnia-driven impairment of alveolar edema resolution due to the high CO_2_-driven downregulation of PM abundance and function of NKA and ENaC [14,38].

We have previously reported that hypercapnia rapidly (within minutes) decreases NKA PM abundance by inducing phosphorylation of the α-subunit of the transporter at the Ser18 residue by protein kinase C- ζ that is activated by a CO_2_-specific signaling pathway including subsequent phosphorylation of extracellular signal-regulated kinase and AMP-activated protein kinase, whereas the c-Jun N terminal kinase drives NKA retrieval from the cell surface [14,15,16,38]. In contrast, potential effects of elevated CO_2_ levels on the regulatory β-subunit of NKA have not been previously reported. In the current study, we describe a novel mechanism by which sustained hypercapnia leads to misfolding of NKA-β in the ER. The NKA-β is a glycoprotein with a molecular mass depending on its glycosylation profile of ~35–55 kDa. Once being synthetized by the ribosomes, the nascent NKA-β protein is co-translationally transported to the ER, where high mannose N-glycan subunit forms are generated. In the ER, the NKA-β undergoes folding, posttranslational modifications, and assembling with the NKA-α. Subsequent maturation and further glycosylation of NKA-β results in the formation of complex-type N-glycan forms, which are located in the Golgi and at the PM [20]. The ER maturation steps play a pivotal role in the NKA-β glycosylation processing [19,21] as well as in the formation of the α:β complexes, which underline the pivotal role of NKA-β in trafficking and the correct membrane insertion of the NKA α-subunit [19,39].

We first investigated whether hypercapnia affects protein levels of NKA-β focusing on the above-mentioned glycosylation states in AEC. We observed a marked time-dependent and dose-dependent increase in the ER-resident high mannose glycosylation forms of NKA-β peaking at 12 h upon exposure to elevated CO_2_ levels. Of note, these effects were independent of extracellular acidosis, which suggests that the observed effects were directly mediated by CO_2_. This increase in ER-resident NKA-β forms was paralleled by a decreased PM abundance and activity of NKA-α:β. Moreover, while the levels of NKA-β were markedly increased in the ER where there was an enhanced interaction of NKA-β with the ER-resident chaperons, calnexin and BiP was evident and we observed a significant decrease in Golgi-resident forms of NKA. This is in line with previous reports that show that mutations in the NKA-α:β interaction regions [19], lipid peroxidation, and activation of oxidative stress by cadmium [20], removal of NKA-β glycosylation sites [20], and overexpression of unassembled NKA β-subunits [40] result in ER retention and increased binding of NKA-β to ER-resident chaperones [20,40]. Since the catalytic NKA-α-subunit cannot leave the ER (and, thus, reach the cellular surface) without being assembled with the regulatory NKA-β-subunit at a 1:1 stoichiometric ratio [19,20] and hypercapnia disturbs normal folding of NKA-β in the ER, which prevents its subsequent further maturation, collectively, our data suggest that elevated CO_2_ levels disrupt the formation of the NKA-α:β complex in the ER, which impairs its delivery to the PM.

Changes in the ER environment or modifications of the protein structure may result in misfolding and protein retention in the ER [41]. It has been shown that high Ca^2+^ and sufficient ATP levels as well as a tightly regulated oxidizing environment are pivotal determinants of proper ER protein folding, glycosylating, and interaction with chaperones [42,43]. Thus, we hypothesized that the ER retention of NKA-β was due to changes in the ER environment. Of note, we establish, in this case, that elevated CO_2_ alters the oxidizing environment of the ER and leads to protein carbonylation. Irreversible attachment of carbonyl groups to proteins is characteristic for a variety of oxidative pathways [44,45] that may promote protein misfolding and lead to various disease states [46,47]. The NKA-β contains a specific cysteine residue (Cys46) in its transmembrane domain that has been described to be highly susceptible to oxidative stress induced by glutathionylation [48], which possibly explains our findings regarding the enhanced ER retention of NKA-β. In addition, carbonylation of ER-resident chaperons may disrupt normal protein folding [46,49]. Whether hypercapnia leads to carbonylation of NKA-β at Cys46 and/or drives carbonylation of the above-mentioned ER-resident chaperons will need to be further investigated in future research. Furthermore, a common cellular response to transcriptional or translation errors is activation of protein carbonylation that “tags” peptides and, thereby, promotes their degradation [50]. Whether hypercapnia induces degradation of carbonylated proteins and NKA-β is currently being investigated in our laboratory. 

In line with our previous findings in a different setting showing that elevated CO_2_ reduces ATP production by inhibiting IDH2 in the TCA cycle and leads to mitochondrial dysfunction [24], our current study shows that sustained hypercapnia markedly decreases intracellular ATP levels. As mentioned above, normal mitochondrial function and sufficient ATP supply of the ER are essential for normal protein folding [23]. We next aimed to study the potential connection between NKA-β oxidation and ER retention upon hypercapnia and the metabolic status of the cell. To overcome the elevated CO_2_-induced suppression of the TCA cycle, we treated AEC with α-KG (a TCA intermediate metabolite) that we have previously shown to rescue ATP production and cellular proliferation in CO_2_ exposed cells [24]. We observed that hypercapnia-induced ER oxidation is significantly reduced after α-KG treatment, which leads to decreased ER-retained NKA-β and increased NKA cell surface abundance and function. Together with our previously published observation, we propose that α-KG treatment may rescue IDH2 activity and, thus, ATP production. Therefore, this decreases ER oxidation and stabilizes normal NKA trafficking. Of note, recent findings suggest that the mechanism by which α-KG operates is not limited to the TCA cycle but may involve various other metabolic and cellular signaling pathways regulating cellular energy supply donor, epigenetics, and prolyl hydroxylases activity [51,52,53]. Thus, elucidation of the exact mechanisms of α-KG action in the context of hypercapnia may need further studies.

Our study has some clear limitations. Although we were able to show that elevated CO_2_ levels induce ER retention of the NKA-β subunit for up to 24 h that may lead to decreased cell surface abundance and activity of the enzyme, it is also evident that, at later time-points (36–72 h of high CO_2_ exposure), elevated levels of NKA-β in the ER are not evident. Our preliminary data (not shown) suggest that this is due to degradation of ER-retained NKA-β. A further study addressing this point is currently a major focus of our laboratory. Additionally, while we demonstrate that, during hypercapnia, an increase in NKA-β is associated with a decrease of the levels of NKA-β in the Golgi apparatus and at the PM, to further strengthen our hypothesis, subsequent studies will be necessary to directly assess trafficking events of both NKA-β and NKA-α from the ER to Golgi and PM. Furthermore, although we demonstrate that ER retention of NKA-β is due to the increased oxidation, we do not know what the primary cause of these reactions is. In addition, despite showing that the formation of carbonyl groups plays a central role in the CO_2_-induced protein oxidation reaction, the potential involvement of other oxidation reactions such as thiol oxidation, glutathionylation, or aromatic hydroxylation has not been investigated. Lastly, it will be important that future studies assess the effects of hypercapnia in more complex systems, such as precision-cut lung slices or human alveolar organoids to further enhance the translational relevance of these findings.

Taken together, our study shows for the first time that sustained hypercapnia decreases NKA cell surface abundance and function by inducing ER retention of its β-subunit via oxidative modification, which prevents its assembly with NKA-α. Furthermore, treatment with α-KG that increases ATP production and rescues the high CO_2_-induced mitochondrial dysfunction attenuates protein oxidation in the ER and, thus, prevents ER retention of the NKA-β and enhances PM abundance and activity of NKA. Since NKA is both a key driver of alveolar fluid clearance and also a central cell adhesion molecule in the alveolar epithelium that is pivotal for prevention of alveolar edema formation, these novel findings may lead to novel therapeutic options that improve resolution of alveolar edema and restores alveolar epithelial barrier function in patients with ARDS and hypercapnia.

## 4. Materials and Methods 

### 4.1. Cell Culture

Primary rat alveolar epithelial type II (ATII) cells were isolated as previously described [14]. Isolation of ATII cells from rats was conducted according to the legal regulations of the German Animal Welfare Act and was approved by the regional authority (Regierungspräsidium Gießen, reference number A12/2013 from March 28, 2013 and reference number G41/2018 from July 17, 2018) of the State of Hessen, Germany. ATII, human alveolar epithelial A549 (ATCC, CCL 185), and A549 cells stably expressing Na,K-ATPase α_1_-subunit fused to a green fluorescent protein (A549-α_1_-GFP) were grown in Dulbecco’s modified Eagle’s media (DMEM, Thermo Fisher Scientific, Darmstadt, Germany) supplemented with 10% fetal bovine serum (FBS, PAA Laboratories, Egelsbach, Germany), 100 U/mL penicillin, and 100 µg/mL streptomycin, as previously described [14]. For experiments, 600,000 A549 or 2,000,000 ATII cells were grown on 60-mm culture dishes (Sarstedt, Nümbrecht, Germany) in 4 mL of culture media. Alternatively, 200,000 A549 or 1,500,000 ATII cells were seeded on permeable membrane supports (BD Falcon, Heidelberg, Germany) with maintaining a liquid/liquid interface. Subconfluent cell monolayers were used for experiments. Cells were incubated in a humidified atmosphere of 5% CO_2_ and 95% air at 37 °C.

### 4.2. CO_2_ Exposure 

ATII and A549 cells were exposed to 40 (normocapnia, Ctrl) or 120 mmHg (hypercapnia, CO_2_) CO_2_ conditions unless otherwise indicated. Normocapnia and hypercapnia media solutions were prepared freshly with DMEM or DMEM/F12, Ham’s F12, and MOPS base to obtain a final pH of 7.4 at 40 (5%) or 120 mmHg (15%) of CO_2_ while maintaining 21% O_2_ balanced with N_2_ and were kept overnight in the humidified C-chamber (BioSpherix Ltd., Parish, NY, USA), as described previously [24]. Levels of CO_2_ in the chamber were controlled by an RO-CO_2_ carbon dioxide controller (BioSpherix Ltd., NY, USA). Before experiments media pH, pCO_2_ and pO_2_ levels were measured using a Rapid-lab blood gas analyzer (Siemens, Erlangen, Germany).

### 4.3. Western Blot Analysis 

Protein concentrations were determined by using the Bradford assay after lysing the cells. Equal protein concentrations were resolved in 7%–10% polyacrylamide gels and transferred to a nitrocellulose membrane using a semidry apparatus from Bio-Rad (Hercules, Berkeley, CA, USA). Membranes were blocked for one hour in 5% fat-free milk and were subsequently incubated with primary antibodies overnight at 4 °C. For some experiments, loading control was performed by staining of the nitrocellulose membranes with Coomassie brilliant blue 250 (Sigma Aldrich, St. Louis, MO, USA). Densitometric analyses were performed by using the Image J software (NIH, Bethesda, MD, USA). 

### 4.4. Cell Surface Biotinylation

After hypercapnia exposure, PM surface proteins were labeled for 20 min by using 1 mg/mL EZ-Link-NHS-SS-biotin (Pierce Biotechnology, Waltham, MA, USA). Proteins were pulled down with streptavidin-agarose beads (Pierce Biotechnology, Waltham, MA, USA) and analyzed by SDS-PAGE and immunoblotting, as described previously [14].

### 4.5. Co-Immunoprecipitation

To assess protein-protein interactions, co-immunoprecipitation was used. AEC were exposed to normocapnia or hypercapnia, washed in PBS, and lysed in immunoprecipitation lysis buffer (20 mM HEPES pH 7.4, 150 mM NaCl, 0.5% NP-40, 2 mM EDTA, 2 mM EGTA, 5% glycerol, protease and phosphatase inhibitors). Afterward, cells were scraped and centrifuged and cell lysates containing 150–300 µg of proteins were incubated with specific antibodies for Na,K-ATPase subunits, GFP, 2,4-DNPH, or IgG negative control and A/G agarose beads (Santa Cruz, Heidelberg, Germany) overnight at 4 °C. Precipitated complexes were eluted from the beads by adding 2X Laemmli buffer, heated at 60 °C for 20 min, and then subjected to SDS-PAGE and Western immunoblotting.

### 4.6. Antibodies and Chemical Compounds

The following antibodies and reagents were used: mouse anti-Na,K-ATPase β-subunit (clone M17-P5-F11) (Thermo Scientific, Rockford, IL, USA), rabbit anti-GFP (Santa Cruz Biotechnology, Dallas, TX, USA), mouse anti-Na,K-ATPase α-subunit (Merck Millipore, Darmstadt, Germany), rabbit anti-β-actin (Sigma Aldrich, St. Louis, MO, USA), mouse anti-transferrin receptor (Invitrogen, Rockford, IL, USA), rabbit anti-PDI (Cell Signaling, Danvers, MA, USA), rabbit anti-GM130 (Cell Signaling, Danvers, MA, USA), rabbit anti-calnexin (Abcam, Cambridge, UK), rabbit anti-BiP (Cell Signaling, Danvers, MA, USA), HRP-conjugated anti-mouse and anti-rabbit IgG (Cell Signaling, Danvers, MA, USA), and Alexa Fluor 488 and 594 conjugated anti-rabbit, anti-mouse (Thermo Scientific, Eugene, OR, USA). α-ketoglutaric acid was obtained from Sigma Aldrich, St. Louis, MO, USA.

### 4.7. Immunofluorescent Microscopy

After hypercapnia exposure cells were fixed with 4% paraformaldehyde, permeabilized with 0.1% Triton X-100, blocked by 3% bovine serum albumin (BSA), and incubated overnight with primary antibodies at 4 °C. Immunofluorescent images were captured by using a Carl Zeiss Axio Observer Z1 microscope (Carl Zeiss, Wetzlar, Germany). 

### 4.8. Isolation of Total, Cytosolic Endoplasmic Reticulum and Golgi Cellular Fractions

Isolation of ER and Golgi was performed by adapting a previously described protocol [54]. After normocapnic and hypercapnic exposure cells were collected, homogenized, and centrifuged 10 min at 1400× *g* following supernatant centrifugation for 10 min at 15,000× *g*, 4 °C. Next, a small amount of the supernatant was aspirated and labeled as “total protein” and the rest was loaded on a sucrose gradient (2.0 M, 1.5 M, and 1.3 M) and centrifuged for 70 min at 152,000× *g*. Afterward, the upper 1 mL of the solution was withdrawn and labeled as “Cytosol.” The ER fraction was collected from the large band at the interface of the 1.3 M sucrose gradient layer, resuspended in lysis buffer, and centrifuged for 45 min at 126,000× *g*, 4 °C. Afterward, the pellet was collected, resuspended in PBS (pH 7.4), and further analyzed by a Western blot. Since ER and Golgi are morphologically linked and have almost the same density during ultracentrifugation, the obtained ER proteins additionally contained Golgi complexes and, in some experiments, this fraction was named “ER, Golgi”. 

### 4.9. Isolation of Soluble Plasma Membrane Proteins

The soluble fractions of PM proteins were obtained by ultracentrifugation, as previously described [55]. After normocapnia or hypercapnia treatment, cells were detached, homogenized in homogenization buffer (10 mM, 1 mM EDTA, 1 mM EGTA, 100 μg/mL N-tosyl-l-phenylalanine chloromethyl ketone, phosphatase, and protease inhibitors), and centrifuged at 500× *g* at 4 °C. Next, supernatants were collected and centrifuged at 100,000× *g* for 1 h at 4 °C. Afterward, the pellet, which contained the crude membrane fraction, was resuspended in homogenization buffer supplemented 1% Triton X-100 and centrifuged at 100,000× *g* for 30 min at 4 °C. After centrifugation, a supernatant containing a soluble membrane fraction was collected and used for the experiments.

### 4.10. Measurement of Na,K-ATPase Enzymatic Activity

To measure ouabain-sensitive Na,K-ATPase activity, a high-sensitivity ATPase assay kit (Innova Biosciences, Cambridge, United Kingdom) was used, according to the instructions of the manufacturer, and was performed as described previously [55]. After treatment with normocapnia or hypercapnia, cells were detached, homogenized, and the PM fraction was isolated. To measure ouabain-sensitive ATPase activity, ouabain was applied to the soluble PM fractions and absorbance, which was measured at 600 nm by an Infinite M200 Pro reader (TECAN, Männedorf, Switzerland). Na,K-ATPase specific activity was calculated by subtraction of ouabain-sensitive from total ATPase activity.

### 4.11. Detection of the Protein Oxidation

Total protein oxidation was measured by using the OxyBlot protein oxidation detection kit (Merck Millipore, Darmstadt, Germany), according to instructions of the manufacturer. After normocapnia or hypercapnia treatment, cells were lysed, and an equal amount of proteins were derivatized by adding 1X 2,4-dinitrophenylhydrazine (DNPH) solution for 15 min. Afterward, a neutralization solution was added, samples were subjected to SDS-PAGE and transferred to nitrocellulose membranes. After incubation with antibodies from the kit, protein oxidation was determined by chemiluminescence and the density of detected protein oxidation was calculated by using the Image J software and normalized to the total protein amounts assessed by Coomassie staining.

### 4.12. Measurement of Intracellular ATP Levels

ATP levels after hypercapnia exposure were measured by an ATP bioluminescence assay kit HSII (Roche Diagnostics, Mannheim, Germany) following the instructions of the manufacturer. Cells were treated with normal or elevated CO_2_ levels, detached, and lysed in the lysis buffer provided with the kit. Next, samples were centrifuged at 10,000× *g* for 60 s and the supernatant was transferred to a fresh tube. Lastly, 50 µL of supernatants were transferred to 96-well plates. Additionally, 50 µL of the luciferase reagent was added to the samples and luminescence was measured immediately by Infinite M200 Pro reader. The obtained values were normalized to the protein amount as assessed by the Bradford assay.

### 4.13. Measurement of Cellular Viability

Cell viability was assessed by using the CASY cell counter and analyzer model TT (Roche Innovatis AG, Reutlingen, Germany) following the instructions of the manufacturer, as determined by an automatized measurement of plasma membrane integrity via an electric impulse. After exposure to normocapnic or hypercapnic conditions, cells were detached by incubation with 0.25% trypsin-EDTA, resuspended in culture medium, diluted in CASY tone solution, and the membrane integrity measurement was performed.

### 4.14. Statistics

Data are presented as mean ± SD unless otherwise indicated. Comparison of two groups was performed by paired (dependent) or unpaired (independent) two-tailed Student’s t-test. Comparisons between more than two groups were performed by one-way or two-way analysis of variance (ANOVA) with a Dunnet test for multiple comparisons. For statistical analysis and data visualization, GraphPad Prism 6 (GraphPad Software, San Diego, CA, USA) was used. A *p*-value of <0.05 was considered to be statistically significant. 

## Abbreviations

α-KG	α-ketoglutaric acid
AEC	alveolar epithelial cell
ALI	acute lung injury
ARDS	acute respiratory distress syndrome
ATII	alveolar epithelial type II
BiP	binding immunoglobulin protein
CO_2_	carbon dioxide
ENaC	epithelial sodium channel
ER	endoplasmic reticulum
GFP	green fluorescent protein
IDH2	isocitrate dehydrogenase 2
NKA	Na,K-ATPase
PM	plasma membrane
TCA	tricarboxylic acid
TfR	transferrin receptor
